# Challenges in Burn Care during the COVID-19 Pandemic—A Scoping Review

**DOI:** 10.3390/jcm11123410

**Published:** 2022-06-14

**Authors:** Michael Kohlhauser, Hanna Luze, Sebastian Philipp Nischwitz, Lars-Peter Kamolz

**Affiliations:** 1 Division of Plastic, Aesthetic and Reconstructive Surgery, Department of Surgery, Medical University of Graz, 8036 Graz, Austria; hanna.luze@medunigraz.at (H.L.); sebastian.nischwitz@medunigraz.at (S.P.N.); lars.kamolz@medunigraz.at (L.-P.K.); 2 COREMED—Cooperative Centre for Regenerative Medicine, Joanneum Research Forschungsgesellschaft mbH, 8010 Graz, Austria

**Keywords:** COVID-19, SARS-CoV-2, burns, burn units, burn care, emergency surgery, pandemic, crisis management

## Abstract

***Objective:*** The aim of this review is to map and summarize the experiences of various burn centers worldwide during the COVID-19 pandemic, in order to enable future strategies with regard to the most effective measures in burn care during pandemics and to detect possible gaps in knowledge. ***Background:*** The coronavirus disease 2019 (COVID-19) pandemic had a major impact on economies, social interactions, and health systems worldwide. Burn units all over the world face a new challenge in maintaining the care of acute burn wounds and follow-up treatments while dealing with constantly changing regulations. Infrastructural changes, the establishment of efficient triage systems, protective measures, personnel resources, in addition to the maintenance of efficient patient care and the guarantee of supply chains, are challenging tasks to be addressed. This review provides an overview of recent developments regarding different strategies and methods used by burn units worldwide to safely overcome the COVID-19 pandemic outbreak. ***Methods:*** A scoping review of the literature was conducted using the electronic databases PubMed and Google Scholar. Publications were screened for the following key terms: burns, burn injuries, thermal injuries, burn center, burn unit, burn ward, in combination with COVID-19, COVID-19 pandemic, SARS-CoV-2, Corona, and Coronavirus. Articles dealing with the management of burn units during the pandemic were further analyzed and included. ***Results:*** Of the 136 publications, 10 were considered relevant to the key question and were included in the present review. Results were divided into six major topics, such as infrastructural and personnel management, triaging, severe burns and emergencies, elective surgeries, patient and visitor management, and outpatient management. ***Conclusion:*** Only a few studies about managing burn units during the COVID-19 pandemic have been published. Personnel resources and equipment needed to be redistributed to cope with country-specific challenges during the COVID-19 pandemic and to maintain adequate burn care. Since all of these articles refer to the period of the initial outbreak, a lack of clinical studies exists regarding the prevention measures taken by burn units during the COVID-19 pandemic. In addition, we identified gaps in knowledge about the impact of implemented measures on burn patient outcomes in the published literature. Further studies are mandatory in order to provide generally applicable guidelines regarding COVID-19 prevention measures at a burn unit.

## 1. Introduction

Hardly any topic has been more present in recent years than the coronavirus disease 2019 (COVID-19), caused by severe acute respiratory syndrome coronavirus 2 (SARS-CoV-2). The first cases originated in Wuhan, the capital of the Hubei province in China, in late 2019. Within a very short time, SARS-CoV-2 rapidly spread and caused a global pandemic [1,2]. COVID-19 can present as asymptomatic on the one hand, and as severe respiratory disorders, and ultimately death on the other. In particular, the transmission of SARS-CoV-2 from asymptomatic people is a major public health concern [2]. To reduce the rate of spread and prevent the collapse of health and socioeconomic systems, pandemic strategies and lockdowns have been established all over the world. Physical distancing, compliance with hygienic measures, rapid testing, and contact tracing, as well as effective protective equipment, are considered powerful methods of preventing a progressive spread of the disease [3]. As a result, members of health professions were exposed not only to physical but also psychological burdens [4,5].

A particular challenge was to provide exigent health care for non-COVID-19 related diseases and emergencies. Burns represent acute traumas that require immediate medical care. In particular, the initial treatment of severely burned patients is a demanding challenge. After the initial treatment, severe burns often require long-term stationary treatments and follow-ups. Consequently, infrastructural modifications, prevention measures, pandemic strategies, and contingency plans for burn units must be defined in order to maintain adequate health care for burn patients during the pandemic. For instance, these could comprise of the setup of triage units and isolation areas, the performance of emergency-only surgeries and the postponement of elective ones. These means should help to prevent the spread of SARS-CoV-2 and comply with burn care, as well as establish and maintain capacities for COVID-19 patients [6,7,8,9,10,11,12,13,14,15]. The aim of this review is to systematically map and summarize the experiences of various burn centers worldwide during the COVID-19 pandemic, along the range of strategies adopted during this time, as well as to identify any existing gaps in knowledge. In Conclusions, this review will enable future policy making with regard to the most effective measures to be taken in burn centers during pandemics.

## 2. Methods

This scoping review was conducted using the methodological guidance of Levac et al. [16] and Peters et al. [17]. Levac et al. [16] recommend the following key phases: (1) identifying the research question; (2) identifying relevant studies; (3) study selection; (4) charting the data; and (5) collating, summarizing, and reporting results. The report of our scoping review was compiled according to PRISMA (Preferred Reporting Items for Systematic Reviews and Meta-Analyses Protocols) guidelines for scoping reviews [18].

### 2.1. Identifying the Research Question

The fundamental research question was formulated with the PCC (population, concept, and context) framework by the Joanna Briggs Institute and recommended by Peters et al. [17,19] (Table 1), as follows: What are the main management challenges in burn units during the COVID-19 pandemic?

### 2.2. Search Strategy to Identify Relevant Studies

A scoping review of the literature, containing information about burn care during the COVID-19 pandemic and published up to 28 January 2022, was performed, using the online databases PubMed and Google Scholar. The applied search strategy included the key terms: (burns[MeSH Terms]) OR (burn injuries[MeSH Terms]) OR (burn center[MeSH Terms]) OR (burn ward[MeSH Terms]) OR (thermal injuries[MeSH Terms]) AND (“COVID-19” OR “COVID-19 pandemic” OR “SARS-CoV-2” OR “Corona” OR “Coronavirus”).

### 2.3. Study Selection

The management of burn units during the COVID-19 pandemic was determined as the fundamental inclusion criterion. First, all search results were exported into Mendeley Desktop (Version 1.19.8) and duplicates were eliminated manually by a researcher. In the next step, titles, abstracts, and later, full-text articles, were analyzed in relation to the fundamental inclusion criterion. To ensure no inequity by wrongful exclusion, this analysis was performed by two investigators. If a consensus between the two researchers was found, a publication was included in the review process. Only full-text original articles published in the English language were eligible. Review articles, comments, and letters were also excluded, with the exception of [9], which described the burn unit strategy during the COVID-19 pandemic at the National Reference Burn Center of Mohammed Vth Military Hospital in Raba during the COVID-19 pandemic and therefore served the purpose of an original article.

### 2.4. Data Charting

A data-charting form was developed by two reviewers to determine which information to include in this scoping review. The two reviewers independently charted the information, discussed the results and continuously updated the data-charting form. Altogether, six major topics could be identified within the eligible publications: infrastructural and personnel management, triaging, severe burns and emergencies, elective surgeries, patient and visitor management, and outpatient management.

## 3. Results

**Figure 1:**
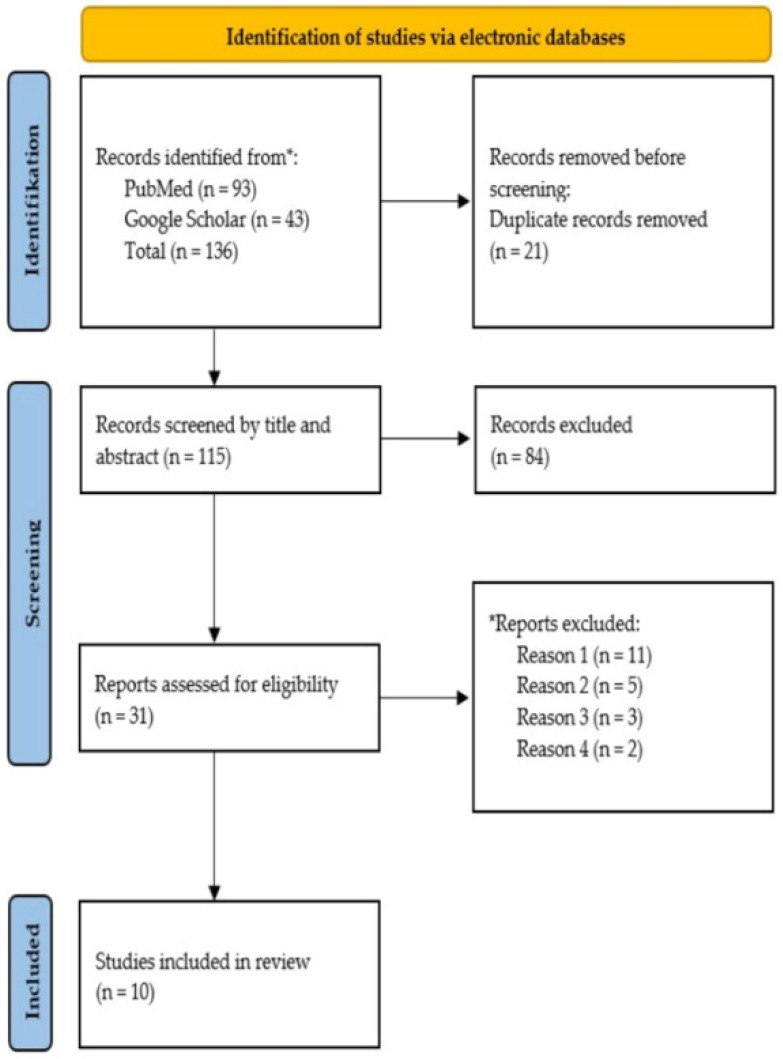
Flow diagram (Preferred Reporting Items for Systematic Reviews and Meta-Analyses—PRISMA) of the study inclusion process. * Reports excluded: Reason 1: no or inadequate focus on management of burn units during the COVID-19 pandemic; Reason 2: not considered to be original quantitative research; Reason 3: unavailable in the English language; Reason 4: irretrievable.

In total, 93 and 43 publications were identified on PubMed and Google Scholar, respectively (*n* = 136). After the elimination of duplicates (*n* = 21), a total of 115 publications were identified from searches of electronic databases and the resulting literature was manually screened for relevant publications. Based on titles and abstracts, 84 were excluded, with 31 full-text articles being retrieved and assessed for eligibility. Of these, 21 articles were excluded for the following reasons: 11 had no or inadequate focus on management of burn units during the COVID-19 pandemic, 5 were not considered to be original quantitative research (e.g., review articles, comments, etc.), 3 were unavailable in the English language, and 2 were irretrievable. Consequently, 10 publications fulfilled the inclusion criterion. The study’s inclusion process is displayed in Figure 1. 

The respective studies originated in Graz, Austria [6]; Chongqing, China [7]; Chelmsford, UK [8]; Rabat, Morocco [9]; Barcelona, Spain; Singapore, Singapore; Torino, Italy; Iowa City and Seattle, USA; Chongqing and Shanghai, China; Birmingham, UK; Tokyo, Japan [10]; Petah Tikva, Israel [11]; Shanghai, China [12]; Johannesburg, Cape Town, Kimberley, and Durban, South Africa [13]; and Delhi, India [14,15]. A brief overview of the included studies and their key points is mentioned in Table 2.

### 3.1. Infrastructural and Personnel Management

#### 3.1.1. Implementation of Separate Areas and Transmission Prevention

To prevent interpatient transmission, the establishment and declaration of different patient rooms and even wards for COVID-19 negative, positive, and suspected cases is of utmost importance [6,7,9,11,12,13,15]. According to Li et al., it is recommended that clearly differentiated areas are designated in order to avoid unnecessary contact [7]. In Mohammed Vth Teaching Armed Forces Hospital in Morocco, three separate pathways to different corridors were created to divide COVID-19 positive, negative, and suspected cases [9]. The only burn unit in Singapore, located at the General Hospital, established a separate isolation wing with the capacity to isolate patients, including intensive care unit (ICU) rooms, a separate operating theater, and support rooms [10]. At the burn unit of the Vardhman Mahavir Medical College and Safdarjung Hospital in Delhi, a single entry and exit system was established, manned by a guard, who was additionally responsible for the control of patient prevention measures, e.g., social distancing, compliance of mask wearing, or hand disinfection [15]. Ma et al. recommended the daily disinfection of wards, as well as outpatient and emergency areas, after finishing clinical work [12].

In addition to the restructuring of existing units, several hospitals and/or wards were fully converted into COVID-19 areas or ICUs to create capacity for infected patients [10,15]. For instance, at the university hospital in Birmingham, operating rooms and some burn ICU beds were reconstructed to provide more capacity for COVID-19 positive patients [10]. Furthermore, hospitals in Barcelona and Seattle fully converted their burn units. As the number of infections increased, additional space was located at the burn ICU of the Harborview Medical Center in Seattle, while the Vall d’Hebron University Hospital in Barcelona fully transformed its burn unit into a non-COVID-19 ICU [10]. In some countries, burn care during the COVID-19 outbreak was centralized in dedicated hospitals [8,10,15]. The university hospital in Birmingham, for example, was designated as a National Burn Centre. Seven rooms at the burn unit were upgraded to fully equipped intensive care rooms to enable the treatment of ventilated burn patients [10].

#### 3.1.2. Equipment and Resources

The most important step in reducing the infection rate and preventing a collapse of the health care systems is the provision of sufficient personal protective equipment (PPE), consisting of masks, preferably FFP2/3 or N95, gloves, protective goggles, and gowns [6,7,8,10,11,12,13,14,15]. In the burn unit of the University Hospital Graz in Austria, PPE was provided and distributed by the hospital management team [6]. In inpatient burn units at the Harborview Medical Center in Seattle, a strategy to economize equipment, mask, and gown reuse, was established during the first months of the pandemic. The available resources were reviewed daily and a reallocation between facilities was carried out to maintain medical capacities [10]. At Southwest Hospital in Chongqing, each ward established a system to register and manage PPE and disinfection supplies. The consumption and inventory of said items was recalculated every day [7,10].

#### 3.1.3. Medical Staff

While Singapore had no need to redistribute personnel resources, in many countries, personnel changes were performed to manage the pandemics’ requirements [10]. Medical staff of burn units in Graz, Petah Tikva, and Barcelona were divided into two teams or, in the case of Delhi, into three, of which only one was present in the hospital at any given time. The teams switched to fortnightly rotas in order to maintain sufficient personnel numbers in the case of positive staff members [6,10,11,15]. In some countries, all surgical staff were appointed to COVID-19 ICUs, leaving only a reduced number of medical staff to maintain vital surgical services during the first infection wave [10]. Only small-scale essential meetings were held face-to-face and these were kept as short as possible. The majority of meetings and also COVID-19 workshops were moved to the Internet, where newly established online platforms were used to exchange information and knowledge [6,7,8,10,12,13]. Outside the hospital, professionals were asked to socially distance, avoid using public transportation and remain in the country during the outbreak [6,7,10]. According to Nischwitz et al., team members at home were asked to prevent infection as much as possible by self-isolating [6]. In the burn unit of Vardhman Mahavir Medical College and Safdarjung Hospital in Delhi, personnel were required to undergo self-quarantine for 14 days after every shift, followed by a COVID-19 test before resuming work [15]. A sign-in and sign-out system for the staff, to track potential transmission, was established in Broomfield Hospital, Chelmsford [8]. In the burn units of Shanghai, staff members had to undergo a temperature measurement before entering the hospital. Only afebrile individuals were allowed to enter the hospital area [12]. According to national guidelines of South Africa, hospital staff must be screened for symptoms daily. Through cellphone-based platforms, quick-screening procedures of burn unit staff were enabled [13]. 

### 3.2. Triaging

Triaging areas were established in front of hospital entrances to screen every non-staff individual for clinical symptoms compatible with a SARS-CoV-2 infection, including body temperature measurement and epidemiological history [6,7,8,9,10,11,12,14,15]. However, triaging methods varied greatly between some burn units. The burn unit of the Rabin Medical Center, Petah Tikva, Israel performed routine COVID-19 tests before hospitalization of patients and their escorts [11], while the burn unit of the All India Institute of Medical Sciences in New Delhi, India only conducted a COVID-19 test in the event of a patient’s suspicious epidemiological history [14]. At the Medical University Graz, Austria, no visitor was required to undergo routine SARS-CoV-2 tests or imaging methods, such as chest X-ray or computed tomography. If necessary, suspected patients, as well as non-responsive ones, were allowed to enter for medical treatment. A SARS-CoV-2 PCR test and an additional chest X-ray in a separate room was then required by those patients, before being transferred to a suspected cases area [6]. Similar practice was performed in the burn unit of the Hospital Universitari Vall d’Hebron in Barcelona [10]. In Southwest Hospital Army Medical University (Chongqing, China), Tokyo Medical University (Tokyo, Japan), and Mohammed Vth Teaching Armed Forces Hospital (Rabat, Morocco), suspected patients were tested by PCR test and chest CT before entering the burn units [9,10]. In addition, all patients admitted to the Mohammed Vth Teaching Armed Forces Hospital (Rabat, Morocco) were required to undergo a laboratory test (blood cell count, Troponin level, LDH, D-Dimers, Procalcitonin, C-reactive protein, urea, and creatinine) [9]. Thorough screening was undertaken in Shanghai and patients with fever and/or an unclear epidemiological history were directed to specialized clinics for suspension of a COVID-19 infection [10,12]. At Ruijin Hospital, Shanghai’s largest hospital, double checks were conducted, at first by phone and then in person prior to entry [10]. If admission was required, a SARS-CoV-2 PCR test and a chest CT were performed in symptomatic patients. In addition, burn units introduced routine COVID-19 tests for children before admission, whereby CT imaging was additionally undertaken for those over six years of age [12]. 

### 3.3. Severe Burns and Emergency

A screening of intubated or non-responsive patients for COVID-19 was not possible by the usual means. According to Kumar et al., the treatment of emergencies should not be delayed by waiting for the result of a COVID-19 test [15]. Some authors announced that if acute surgery was necessary, patients should be treated as COVID-19 confirmed cases [6,7,8,9,14]. In contrast, Yaacobi et al. reported in his paper from October 2020 that Petah Tikva’s strategy had included testing of all patients before surgery, even in emergencies [11]. 

Highly preventative standards and sufficient PPE were used during all emergency interventions, including airway management [8,9,10,11,12,14]. Yaccobi et al. recommended that these treatments should be exclusively performed by minimal staff with complete PPE [11]. In order to prevent the spread of SARS-CoV-2, Saha et al. advised operating on positive patients in a negative-pressure operating theater [14]. Many burn centers isolated emergency patients, who had an unknown COVID-19 status, for up to 14 days [7,10,11]. In Graz, burn patients were transferred from the COVID-19 positive surgery wing to the COVID-19 suspected ICU. They were kept there until two negative PCR tests had been obtained [6]. Furthermore, Chelmsford’s emergency burn patients were treated in a COVID-19 positive ward until proven negative [8].

### 3.4. Elective Surgeries

In the majority of cases, operating theaters were reserved for life-supporting intervention, including severe burns, and elective procedures were postponed and rescheduled [6,7,9,10,11,13,14,15]. In the case of elective surgery, patients were screened before procedure [8,12]. Patients at St. Andrew’s Centre for Plastic Surgery & Burns in Broomfield Hospital who required elective procedures, were required to present a negative PCR test result no more than 72 h prior to surgery. Additionally, these patients were required to self-isolate pre-admission [8].

### 3.5. Patient Management and Visitors

The requirement to wear masks and maintain a safe distance of 2 m between all individuals was established in most hospital areas [6,10]. In Chongqing, an attempt was made to set aside a single room for each patient regardless of their infection status [10]. According to Nischwitz et al. hospitalized patients should be informed of the risk of infection by a written information sheet [6]. Incoming patients at Southwest Hospital Chongqing Army Medical University (China), including pediatric burns, were isolated in a separate room, initially for 14 days. After isolation, patients were transferred to the regular ward with other patients and were allowed to access public areas. Only one caregiver was allowed to stay with adult burn patients, while two were permitted to stay with children [7]. Similar procedures were adopted by burn units in Delhi, where positive patients were isolated for 14 days [14] and only one caregiver per patient was allowed on the burn ward [14,15]. At the Rabin Medical Center, Petah Tikva, it was mandatory that the same person stay with an inpatient pediatric burn patient during the hospitalization period. The patient and the accompanying person were tested before admission and were required to stay together during this time [11]. In Broomfield Hospital, all inpatients had to undergo a COVID-19 swab test before admission, and this was repeated after five days [8]. Some authors recommended the observation and recording of typical symptoms as well as vital signs continuously for all patients [7,10,11]. Saha et al. recommend that patients suffering from COVID-19-associated symptoms during hospitalization should be tested as well as given a chest CT scan [14]. According to the COVID-19 politics of the Department of Surgery at the University Hospital Graz, patients who had developed COVID-19-like symptoms during hospitalization were separated on the same ward and required to undergo a SARS-CoV-2 PCR test. Positive patients were then transferred to a COVID-19 positive ward. A return was possible after two negative test results [6]. In South Africa hospitalized burn patients infected with SARS-CoV-2 were treated according to the severity of burns. Minor burns were treated in the general COVID-19 area, while major burns were transferred to a specialized burn COVID-19 section [13]. In several burn units, short-term dressings were replaced by long-term ones in order to reduce the frequency of change [10,15].

In many hospitals, visitors were minimized and monitored before entry [9,10], with some denied access and given permission to only enter in special cases [6,13]. Morocco allowed one visitor per patient daily, for whom temperature assessment and epidemiological screening before entry were mandatory [9]. In some hospitals, video calls were arranged in place of physical visits [7,10,13,14]. Patients at Vardhman Mahavir Medical College and Safdarjung Hospital in Delhi who required long-term therapy with many in- or outpatient follow up treatments were tested at regular intervals, including post-discharge [15].

### 3.6. Outpatient Management

In Broomfield Hospital, Chelmsford, a one-way system through the department was introduced to reduce patients’ interaction [8]. In addition to the ward, the outpatient department of the burn unit in the Vardhman Mahavir Medical College and Safdarjung Hospital, Delhi, India was divided into two separate areas, one for non-COVID-19 cases and the other for COVID-19 suspected or confirmed cases [15]. At the Department of Surgery at the University Hospital Graz, Austria, essential outpatient appointments were planned with enough time to prevent crowds in waiting rooms. Accompanying persons were also reduced and only allowed if necessary. Waiting areas and other frequented zones were enlarged wherever possible to keep a minimum distance of 2 m between persons [6]. A similar procedure was established in Shanghai burn units [12]. At the Southwest Hospital in Chongqing, most outpatient visits were postponed or cancelled in order to minimize numbers on the hospitals’ premises during the outbreak [7]. If possible, dressing changes of mild burns were outsourced to nearby hospitals or resident doctors [6,7]. At the Queen Elizabeth Hospital, Birmingham, patients with moderate burns were examined by a burn surgeon and were admitted if necessary. Minor burn (<10% total body surface area) patients were provided with enough dressing material for home treatment [10]. Similarly, at the Rabin Medical Center in Petah Tikva, the guardians of pediatric patients with small burn injuries were advised to change wound dressings at home after being instructed about the process [11]. In South Africa similar instructions were given for patients in home isolation [13]. 

In addition to the necessary face-to-face consultations, telemedicine has seen a significant upsurge to maintain the necessary doctor-patient interaction [8,10,11,13,14]. The benefits of telemedicine and telecommunication were also used with recovered burn patients, who could consult medical professionals online by using apps or a phone [7,10,11,12]. Furthermore, patients were given the opportunity to log in every day and receive an update on their rehabilitation status [7]. In South Africa, telemedicine was additionally used by burn experts to guide healthcare practitioners on further patient treatment at the referring hospitals [13]. Outpatient visits at Harborview Medical Center burn unit in Seattle were reduced by 90% with reorganization and telemedicine [10]. 

## 4. Discussion

Due to the uneven distribution regarding the severity of COVID-19 infections, burn units faced different challenges worldwide. Various strategies to maintain burn care during the COVID-19 pandemic have been published thus far. A wide consensus between the publishing hospitals for infrastructural changes was reached; these adaptations are highly sought after by burn units in order that they meet constantly changing requirements to maintain burn care during the pandemic. Depending on the country-specific situation, burn units created various additional capacities for COVID-19 patients. 

In particular, the creation of separate areas for COVID-19 infected patients and additional ICU capacities is necessary in countries with high infection rates. Steps, such as outsourcing suspected patients to special facilities, are only possible in large cities, such as Shanghai [10,12] and Chongqing [7,10], while smaller cities have to consider other options. A separation of COVID-19 positive and COVID-19 suspected patients, as performed in Graz [6], for example, is a reasonable approach to further reduce the spread between patients. 

Similar opinions were also found regarding the use of PPE and disinfection supplies; adequate supply is of utmost importance to reduce spread and maintain medical capacities. In some countries, PPE was provided and distributed by a hospital’s management. The Seattle hospital even had to reuse equipment to conserve stocks [10]. Daily consumption and inventory calculations, as performed in Chongqing, may be a reasonable measure in order to prevent the shortage of PPE and disinfection supplies [7,10].

Due to differences in the spread of COVID-19 and available resources, strategies vary greatly with regard to the deployment of medical personnel. This has also resulted in a large amount of extra work having to be undertaken by the existing medical staff with no extra workforce provision. In Spain, the medical staff at burn units were completely transferred to COVID-19 units to support domestic staff, due to the infection rate, while it was not necessary to redistribute staff in Singapore [10]. Most approaches include multimedia developments, such as virtual meetings, online staff training, and telemedical advice. In particular, the replacement of large face-to-face meetings with virtual ones is an urgently needed step in the prevention of inter-hospital transmission and may also be used in the future.

Consequently, health professionals were affected by new challenges, including frequently changing guidelines and protocols, a multiplicity of critically ill patients, infected colleagues, minimized personnel associated with additional burdens, and tough decisions, as well as the risk of infecting themselves or their loved ones. Thus, caring for patients during the time of COVID-19 is a challenging task for medical staff, not only with an additional workload, but also through increased psychological pressure. Supporting the mental health of medical staff is of utmost importance during the pandemic to prevent negative psychological effects such as anxiety, burnout, depression, and post-traumatic stress disorder. As described by Walton et al., opportunities should be provided at all levels to promote the mental health of medical staff and reduce psychological distress [4]. Meanwhile the COVID-19 crisis has already lasted for more than 2 years. The mental wellbeing of health professionals is, now more than ever, critically important for maintenance of eminent medical care. It is vital that staff members feel supported, protected, and listened to during the pandemic.

Special triaging and screening methods were established all over the world. There are numerous international differences regarding the screening for COVID-19 symptoms and the proof of infection. Yet, due to the lack of novel literature, no generally valid statement concerning the number or the type of screening methods can be made. However, we believe that it is of the utmost importance to use valid methods to safely detect COVID-19 positive patients for further measures in order to prevent spread.

Reducing the number of patients treated is another step in the efficient use of personnel and infrastructural resources, and reduction in further spread of the disease. The estimation of required resources was a difficult task, particularly during the outbreak. With the exception of Smith et al. [8] and Ma et al. [12], all other authors describe the cancellation and postponement of elective interventions during the COVID-19 outbreak in order to save resources and capacities for emergencies.

During the COVID-19 outbreak, management of outpatient burn patients was also modified to reduce the risk of inter-hospital spread with the adoption of several social distancing measures. Most outpatient appointments were cancelled and only essential ones were performed under certain preventative measures [6,8,10,12]. Instead, online consultation systems were adopted by various burn units to maintain referrals and follow-ups [7,8,10,11,12,13,14]. Minor burn patients and their loved ones were advised by some burn units to perform their dressing changes at home [10,11]. Although these measures are certainly a useful approach to minimizing the transmission of SARS-CoV-2, it remains unclear how they affect the treatment of burn patients. No data on the impact of these measures on burn patient outcomes are available in the published literature so far. In order to make a valid statement, publications reporting the impact of these measures on the treatment outcome of burn care are indispensable.

Given the expedient nature of major burns, which require immediate medical care, social distancing or the avoidance of direct patient contact is not a viable option. In particular, medical personnel performing laryngoscopy, intubations, and bronchoscopy, which are essential in the treatment of severe burns, are exposed to a large number of aerosols associated with an increased risk of infection [8,11,13,14]. Therefore, the establishment of specific protocols, the availability of adequate PPE, extensive staff training, and infrastructural adjustments are mandatory [20]. The correct behavior of personnel through specific protocols and training will prevent misconduct, thereby reducing the risk of infection and thus the spread of SARS-CoV-2.

Using these efficient measures to prevent the spread of COVID-19, coping with the pandemic was feasible all over the world. Within a very short time, triaging and infrastructural changes, including the postponement or cancellation of elective surgeries, were established. The isolation of symptomatic cases was used as an early COVID-19 containment measure. However, through continuous improvement of the capacity and duration of swab tests, a strategy change from isolation to swab testing was recognized. Consequently, COVID-19 swab testing emerged as an effective measure, which was incorporated into the prevention strategy of many hospitals, and was proven to be an important step in the rebooting of health care systems.

Rebooting the system and conducting elective interventions proves to be much more difficult. Only a single article reports measures to re-establish a burn units’ management process during the pandemic, a necessary strategy during and after the decrease of infection rates [6]. While systems have slowly restarted, adaptations to the proven measures are continually being established. Postponed surgeries have to be rescheduled according to priority and processed by taking into account the available capacity.

It is necessary to keep the capacity balanced in order to maintain resources in the event of another infection wave. Notably, the recognition of asymptomatic individuals is of highest priority. Therefore, rapid antigen tests to identify SARS-CoV-2 infected individuals with high viral loads, the usage of nose-mouth masks, and safety distancing play a key role in the hospital setup to maintain burn care provision during the pandemic. The real-time reverse transcription PCR technology, with high specificity and sensitivity, is still considered the gold standard in the diagnosis of SARS-CoV-2 infections, but it requires professional expertise, expensive reagents, specialized equipment, and a copious amount of time. Antigen tests are an inexpensive and rapid alternative to PCR methods, which enables frequent usage [21,22]. Toptan et al. demonstrated a high correlation (88.2–89.6%) between PCR and antigen tests in samples with high viral loads, which are associated with lower threshold cycle (cT) values [21]. Therefore, antigen tests have demonstrated more efficiency within the first days of a COVID-19 infection since the viral load is particularly high within that period of time [22]. The use of antigen tests enables a large number of patients to be tested expeditiously. Due to a minimized evaluation period, a more favorable separation of COVID-19 positive and negative patients is possible, and the requirement of isolation areas for suspected cases, which played a key role during the first wave, can almost be completely eliminated. The high availability and low cost of antigen tests enables routine tests to be obviated. All staff members at the Division of Plastic, Aesthetic and Reconstructive Surgery at the University Hospital Graz have to routinely undergo COVID-19 antigen tests. Suspected results are checked using PCR methods. The aim is to identify asymptomatic cases among the medical staff and prevent further transmission.

Another eminent measure to maintain medical care, including the treatment of burns, is the continuous wearing of a mouth-nose mask by health professionals to prevent the spread of SARS-CoV-2, and thus staff absence [6,12]. While N95 masks block at least 95% of aerosol particles, the filter efficiency of FFP1, FFP2, and FFP3 masks is 80%, 94%, and 99%, respectively [12]. However, wearing face masks for several hours causes undesirable side effects, including upper and lower respiratory disorders, dry and irritated eyes, headache, sleep disturbances, concentration difficulties, and overall reduction of job performance [13,14]. Maniaci et al. demonstrated a significant reduction of these disorders due to the introduction of several easily applicable best practice measures (e.g., smoking abstinence, fresh air breaks, isotonic nasal wash, and single mask use) [13]. Since members of health care professions have a particularly onerous responsibility and masks are indispensable during this challenging time, easily implementable countermeasures, such as the best practice measures presented, are a feasible option for reducing physical and psychological disorders and increasing job performance.

COVID-19 and its transmission is a dynamic process, which leads to rapid and unforeseeable occurrences. In general, an exact overview is difficult to specify, due to the rapid changes in prevention measures and hospital management. All of the discussed strategies refer to the period of the initial outbreak during the first half of 2020, mostly combatting the reconstruction of personnel and infrastructural resources and adequate supply of required equipment to conquer the COVID-19 pandemic. Although some burn units have reported their measures [6,7,8,9,10,11,12,13,14,15], there are no data on the impact of these with regard to treatment outcomes. Most of the data published in the literature thus far refer to epidemiological and demographic data in addition to injury and treatment characteristics of burn patients [8,15,23,24,25,26,27,28,29,30,31].

Meanwhile, it is well-known that the COVID 19 pandemic occurs in waves, in which the number of infections constantly fluctuates. Although the desired COVID-19 vaccines were developed a year ago, COVID-19 spreads indefatigably and reaches new records of infections with every ensuing wave. Over the last few months, the incidence of COVID-19 has increased exponentially with the appearance of the novel omicron variant. While the number of confirmed cases in the fourth wave reached a high of 4,584,765 on 16 August 2021, the pandemic peaked in the fifth wave with 23,305,707 confirmed cases on 24 January 2022 [32]. The novel omicron variant of SARS-CoV-2 has less impact on health compared with previous COVID-19 waves because of increased levels of population immunity and the possibility of reduced severity in omicron infections. However, 21% of hospitalized patients infected with the SARS-CoV-2 omicron variant in South Africa, which is known for its generally young population, showed severe symptoms [33]. Thus, proper preventive measures and management strategies are an important part of maintaining the health care system and ensuring an ordinary hospital workflow. 

Despite this increase in COVID-19 infections, no literature on burn center measures and strategies or their outcomes during the current wave of the pandemic is available. Nevertheless, novel publications about these adaptations and their impact on patient treatment outcomes are vital in order to enable an international exchange to address the ongoing challenges of providing continuous burn care during the pandemic. Until these publications are available, burn units are dependent upon country-specific recommendations regarding infection rates, as well as requisite features and components of treatment processes. 

Our scoping review has some inherent limitations. For one, no global analysis can currently be conducted due to a scarcity of relevant articles, relating to the prevention tactics of a particular city. All articles related to the time of the first outbreak and no reports were published about burn unit management during the ongoing pandemic. In addition, we identified gaps in knowledge about the impact of these measures on burn patient outcomes in the available published literature up to the present time. A further addition to this issue is that the COVID-19 pandemic depicts a novel challenge with a very dynamic progression, which leads to a constant development of several modern prevention measures. Finally, our review is limited to articles retrieved from PubMed and Google Scholar with the possibility of missed publications. Despite having two investigators screen the literature, a possible wrongful exclusion cannot be ruled out.

## 5. Conclusions

Various strategies have been presented by burn units during the COVID-19 pandemic. To adapt to country-specific requirements, resources needed to be redistributed and/or extended to maintain adequate burn care. Due to the fact that the currently available publications report country-specific rather than global developments, only focusing on the first wave of the pandemic, a generally applicable strategy may not be defined. However, it has been shown that physical distancing, compliance with hygienic measures, rapid testing and the use of adequate protective equipment are considered powerful methods of preventing a progressive spread of the disease. In order to overcome the pandemic and to define useful elements that may be included in the countries’ own strategies, international exchange of information is of utmost importance. However, due to the variability in the trend of the COVID-19 pandemic, no general guidelines or recommendations are available and permanent adaptations are necessary. Since all the measures described here date back to the first year of the COVID-19 pandemic, with no data about the impact of these modifications on burn treatment outcomes, current publications are urgently required to gauge their validity.

## Figures and Tables

**Table 1:** Creation of the research question according to the PCC framework.

Population	Concept	Context
Burn units	Management challenges	COVID-19 pandemic
What are the main management challenges in burn units during the COVID-19 pandemic?		

**Table 2:** An overview of the eligible studies and their key points.

Study	Date Released	Address of Correspondence	Nation	Study Design	Key Points/Summary
Nischwitz et al. [6]	June 2020	Division of Plastic, Aesthetic and Reconstructive Surgery, Department of Surgery, Medical University of Graz,	Austria	Single center report of prevention measures and reboot strategies	This article gives an overview of the prevention measures, such as the establishment of separate areas for COVID-19 positive and suspected patients, divisions of teams, equipment management, triaging, postponement of elective interventions, ward management, and handling of outpatients. Additionally, the article includes reboot strategies.
Li et al. [7]	April 2020	Institute of Burn Research, State Key Laboratory of Trauma, Burns and Combined Injury, Southwest Hospital, Army Medical University, Chongqing	China	Recommendation	This paper summarizes some management strategies for burn-ward-based experiences at the Southwest Hospital, Army Medical University in Chongqing and the Chinese national and international public health issues, such as ward organization, emergency management, personnel protection and training, and patients and caregivers management, as well as the maintaining of mental health for isolated patients.
Smith et al. [8]	January 2021	St Andrew’s Centre for Plastic Surgery & Burns, Broomfield Hospital, Chelmsford	United Kingdom	Single-center prospective controlled cohort study	This study reviews the measures implemented at St. Andrew’s Centre for Plastic Surgery & Burns, Broomfield Hospital, Chelmsford. Additionally, the study includes demographics, appointments, service satisfaction, and treatment outcomes in the period of April-May 2020.
Fouadi et al. [9]	July 2020	Plastic Reconstructive and Burn Surgery Department; Emergency Department; Stomatology and Maxillo-Facial Surgery Department Mohammed Vth Teaching Armed Forces Hospital	Morocco	Letter to the editor	This paper gives a brief overview of the management strategies of the National Reference Burn Center of Mohammed Vth Military Hospital in Raba during the COVID-19 outbreak, including the establishment of separate pathways and areas, the usage of personal protective equipment, the postponement of elective interventions, patient education for self-dressing changes, triaging, and a reduction of daily visitors.
Barret et al. [10]	April 2020	Department of Plastic Surgery and Burns, Hospital Universitari Vall d’Hebron, Barcelona; Department of Plastic Reconstructive and Aesthetic surgery, Singapore General Hospital; Anaesthesia and Intensive Care, Città della Salute di Torino; Division of Plastics and Reconstructive Surgery, Department of Surgery, University of Iowa Hospitals and Clinics; Institute of Burn Research, Southwest Hospital Army (Third Military) Medical University, Chongqing; University Hospitals Birmingham Foundation Trust, Queen Elizabeth Hospital, Birmingham; Harborview Medical Center, Seattle; Department of Burn and Plastic Surgery, Ruijin Hospital, School of Medicine, Shanghai Jiaotong University, Shangai; Division of Acute Care Surgery, Department of Surgery, University of Iowa Hospitals and Clinics, Iowa; Department of Plastic and Reconstructive Surgery, Tokyo Medical University, Tokyo	Spain, Singapore, Italy, USA, China, United Kingdom, Japan	International multicenter report of prevention measures	This article contains global strategies of infrastructure and personnel management, triaging methods, the management of emergency cases, information about scheduling of elective interventions, outpatient management, and inpatient and visitor strategies. Additionally, some information is given about the pandemic situation in different countries.
Yaacobi et al. [11]	October 2020	Department of Plastic Surgery & Burns, Rabin Medical Center, Petah Tikva	Israel	Retrospective single center study and summary about prevention measures	This article reports prevention strategies for pediatric burns during the COVID-19 outbreak, such as inpatient admission and treatment, outpatient management, and personnel management, as well as surgery and bedside procedure.
Ma et al. [12]	January 2021	Department of Burn Surgery, Changhai Hospital, Navy Military Medical University, Shanghai	China	Summary article	This article presents a summary of measures in Shanghai burn departments for COVID-19 prevention, including in- and outpatient management, telemedicine follow-ups, ward management, and transmission prevention during surgery procedures and emergency cases, as well as triaging.
Ede et al. [13]	February 2021	Chris Hani Baragwanath Academic Hospital, Johannesburg	South Africa	Short report	The article includes strategies and measures, such as the origination of burn units, management of ward work and visitors, guidance of COVID-19 positive burn patients, and management of burn wounds, as well as post-discharge care and rehabilitation in low- and middle-income countries, based on the situation in South Africa during the COVID-19 pandemic.
Saha et al. [14]	May 2020	Department of Plastic Reconstructive and Burns Surgery, Jai Prakash Narayan Apex Trauma Centre, Delhi	India	Summary article	Within this article, the authors summarize guidelines for team training and preserving the workforce, advice for emergency burn clinics, in-hospital treatment of burns, burn surgeries, discharge and follow-up, and leveraging technology during the COVID-19 pandemic.
Kumar et al. [15]	December 2020	Department of Burns, Plastic and Maxillofacial Surgery, Vardhman Mahavir Medical College and Safdarjung Hospital, Delhi	India	Retrospective observational study	The authors give a brief summary of infrastructural adaptations, the establishment of a screening protocol, management of burn injuries, prevention management of healthcare workers, the usage of adequate equipment, and discharge criteria in the burn units of Delhi during the time of COVID-19.
